# Association of Oxidative Stress and Production of Inflammatory Mediators Matrix Metalloproteinase-9 and Interleukin 6: Systemic Events in Radicular Cysts

**DOI:** 10.7759/cureus.7822

**Published:** 2020-04-25

**Authors:** Arif Malik, Sahar Chawla, Ambreen Tauseef, Hira Sohail, Farhat Ijaz, Aamenah Malik, Farzana Rahman, Ghulam Muhammad, Saad Khakwani

**Affiliations:** 1 Biochemistry, Institute of Molecular Biology and Biotechnology, Lahore University, Lahore, PAK; 2 Biochemistry, Combined Military Hospital Lahore Medical College and Institute of Dentistry, Lahore, PAK; 3 Physiology, Combined Military Hospital Lahore Medical College and Institute of Dentistry, Lahore, PAK; 4 Biochemistry, Combined Military Hospital Lahore Medical College and Institute of Dentistry, Lahore, PSE; 5 Orthodontics, Triangle Orthodontics, Chapel Hill, USA; 6 Dentistry: Periodontology, Combined Military Hospital Lahore Medical College and Institute of Dentistry, Lahore, PAK; 7 Dentistry, Combined Military Hospital Lahore Medical College and Institute of Dentistry, Lahore, PAK

**Keywords:** antioxidants, inflammatory mediators, radicular cyst

## Abstract

Background

Matrix metalloproteinase-9 (MMP-9) and antioxidants are associated with the pathogenesis of cysts and may initiate and sustain the formation of new capillaries.

Objective

The objective of this study was to determine the association of oxidative stress and the production of inflammatory mediators MMP-9 and interleukin 6 (IL-6) in systemic events in radicular cyst growth.

Materials and methods

Fifty patients (34 men, 16 women) with periapical granulomas and radicular cysts were included in this cross-sectional study. Twenty subjects (12 men, eight women) with no signs of periodontal diseases were recruited as controls. Blood serum levels of MMP-9, IL-6, superoxide dismutase (SOD), malondialdehyde (MDA), and glutathione peroxidase (GPx) were recorded. We also recorded body mass index (BMI) and tumor necrosis factor-alpha (TNF-alpha) levels.

Results

The mean age of the test group patients and control patients was 45.9 and 48.8 years, respectively. The BMI of test group patients (23.77± 3.88 kg/m^2^) was higher than that of the controls (27.98 ± 3.88 kg/m^2^; p ≤ 0.000). Levels of serum MDA (p ≤ 0.033), IL-6 (p ≤ 0.041), TNF-alpha (p ≤ 0.004), and MMP-9 (p ≤ 0.033) were significantly increased in patients as compared with control values. SOD (p ≤ 0.003) and GPx (p ≤ 0.033) levels were significantly reduced in patients as compared with controls.

Conclusion

Oxidative imbalance and the increased production of inflammatory mediators may be associated with systemic events in radicular cysts. Bone-resorbing mediators and proinflammatory cytokines that were evaluated in the study (MMP-9, IL-6, C-reactive protein, TNF-alpha) were also elevated in the serum of the ailing group, thus documenting a well-established role for these circulating biochemical variables in the course of the progression and pathogenesis of radicular cyst development.

## Introduction

Cyst growth and maturity involves the provision of stimulus by antigens, microbes, fibroblasts, and growth factors that trigger the inflammatory response by releasing various cytokines, ultimately causing explosive cell division. As the epithelial proliferation in a cystic cavity continues, cells in the middle of the mass become deficient of nourishment and begin necrosis. The degenerative tissue and cell debris in the cyst cavity are chemotactic for neutrophils, causing influx into the cyst lumen. The cystic contents have a higher osmotic load as compared to the surrounding tissue serum.

Moreover, the necrotic and degenerating cells in the interior of the cyst cavity discharge an excessive amount of molecular species, further raising the osmotic pressure of the cyst void, resulting in the flow of liquid from tissues into the cyst lumen. The outcome is an elevated intracystic pressure that can result in osteoclastic bone resorption and cyst enlargement [1-3]. A radicular cyst (RC) is a result of chronic inflammation in response to inflammatory mediators created from the necrosis of dental pulp [4]. According to Khot et al., RCs “derive their epithelial lining from the proliferation of small odontogenic epithelial residues within the periodontal ligament,” which contributes to the progression of the lesion [2]. The regulation of extracellular matrix synthesis and degradation is a vital function of metalloproteases (MMPs), which are also involved in embryogenesis, ovulation, development of nerve tissue, vessel formation, apoptosis, and monitoring of various inflammatory mediators, including cytokine cleavage and activation of mediators and defensins. In addition to their involvement in the above-mentioned physiological processes, they are mediators of many infectious and inflammatory diseases, including apical periodontal bone disorders and other periapical lesions [5].

The MMPs predominantly expressed in infectious and necrotic odontogenic lesions are -1, -2, -3, -8, 9, and -13 [6]. Reactive oxygen species (ROS) signaling may activate MMPs like MMP-2 and MMP-9 and inflammatory mediators, especially in periodontal tissues [7].

Oxidants can cause tissue injury via damage to deoxyribonucleic acid (DNA) and proteins, peroxidative injury to lipid membranes, activation of proinflammatory cytokines, and proteases like MMPs. Oxidative stress has an important role in the pathogenesis of apical periodontitis that can lead to RC formation [8]. Antioxidants present in small concentrations, oppose free radical action, and inhibit or delay substrate oxidation [9].

The mode of action of antioxidants involves both non-enzymatic and enzymatic reactions. Primary antioxidants are superoxide dismutase (SOD), glutathione peroxidase (GPx), and malondialdehyde (MDA) [10-11]. GPx and SOD offset the oxidative effects and prevent damage to cellular DNA [12]. MDA is produced via the peroxidation of polyunsaturated fatty acids, and MDA may reduce the activity of glutathione peroxidase in periapical granulomas [13].

SOD removes superoxide radicals by catalyzing the dismutation of molecular oxygen and hydrogen peroxide. GPx is an oxidant enzyme and requires selenium as a cofactor. In cells, the main role of GPx is to scavenge hydrogen peroxide [14].

Peroxide activation increases the levels of interleukin 6 (IL-6), and MMPs can alter the impact of peroxide [15]. The continuous secretion of proinflammatory cytokines in periodontal tissues (e.g., IL-1, IL-2, IL-6, and tumor necrosis factor-alpha (TNF-alpha)) has been observed. Also, reduced levels of regulatory cytokines (e.g., transforming growth factor-beta-1 and IL-10) have been associated with continued inflammation of the ligaments and supporting tooth structures [16].

This cross-sectional study was designed to determine the association of oxidative stress and the production of inflammatory mediators MMP-9 and IL-6 in systemic events in RC growth.

## Materials and methods

Fifty patients (34 men, 16 women) aged 20 to 40 years were recruited from the Department of Dentistry, University College of Dentistry, Lahore. Patients with periapical granulomas and RCs were included in the study. These patients had a clinical attachment loss > 3 mm, probing depth > 5 mm, and bleeding on probing. Radiographic evidence of round, well-defined radiolucencies of periapical tissues was required for inclusion. We excluded patients receiving antibiotic therapy (within three months of the start of the study period), those with compromised periodontal status, pregnant and lactating women, and those suffering from any chronic infection or depression. Our control group consisted of 20 subjects (12 men, eight women) with healthy, attached gingiva, no signs of periodontal disease nor bleeding on probing. Our institutional ethical committee approved the study protocol.

We assessed patient body mass index (BMI) and collected blood samples to evaluate MMP-9, IL-6, SOD, MDA, and GPx levels via a chemical assay. Levels of TNF-alpha and IL-6 were measured via enzyme-linked immunosorbent assay.

Statistical analysis

Study data were analyzed using IBM SPSS Statistics for Windows, Version 21.0 (IBM Corp., Armonk, NY). Variables were shown as mean ± standard deviation (SD). Patient and control values were compared using the student ‘t’ test. P ≤ 0.05 was considered significant.

## Results

The mean ages of patients and controls were 45.9 and 48.8 years, respectively. Mean patient BMI (23.77 ± 3.88 kg/m^2^) were higher than mean control BMI (27.98 ± 3.88 kg/m^2^; p ≤ 0.000). Levels of serum MDA (p ≤ 0.033), IL-6 (p ≤ 0.041), TNF-alpha (p ≤ 0.004), and MMP-9 (p ≤ 0.033) were significantly increased in patients as compared to those in the control group (Table 1). However, the levels of SOD (p ≤ 0.003) and GPx (p ≤ 0.033) were significantly decreased in patients compared to controls.

**Table 1:** Levels of circulating biochemical variables in patients with a periapical cyst MDA, malondialdehyde; SOD, superoxide dismutase; IL-6, interleukin-6; TNF-α, tumor necrosis factor-alpha; MMP9, matrix metalloproteinase 9; GPx, glutathione peroxidase

Variables	Controls (n=20)	Subjects (n=50)	P-value
MDA (nmol/ml)	1.37 ± 0.03	4.11 ± 0.24	0.033
SOD (nmol/ml)	0.11±0.03	0.03±0.01	0.003
GPx (nmol/ml)	7.87±1.88	5.88±1.98	0.033
IL-6 (pg/ml)	4.98±0.55	7.78±1.98	0.041
TNF-α (pg/ml)	27.98±4.78	30.87±5.89	0.004
MMP-9 (pmol)	44.78±3.98	166±6.99	0.033

## Discussion

Periodontitis is distinguished by immune-mediated damage of periodontal supporting tissues and loss of teeth. Apical lesions can progress to form an RC, and the RC’s level of growth is mainly due to the increased levels of MMP-9 [4].

Our findings of significantly increased levels of serum MDA, IL-6, TNF-alpha, and MMP-9 in patients when compared to controls align with a previous study that suggest RC formation may be due to the degradation of collagen via MMPs (i.e., the main stop in the loss of periodontal supporting tissue) [17]. However, another study suggests the primary factor in RC formation is due to collagenases [18]. The separation of epithelium from the connective tissues results in lesion progression and recurrence [19-20]. 

Angiogenesis is associated with the degradation of vascular basement membrane and renovation of the extracellular matrix to allow endothelial cells to travel into the surrounding tissues [21]. MMP-9 may have an important role in the angiogenesis of odontogenic cysts. Also, ROS can directly activate MMP-8 and MMP-9 in periodontal tissues via oxidizing enzymes [22]. Oxidative non-proteolytic activation of MMP seems to be important in periodontal inflammation. The exact mechanism of involving MMP-9 in intracellular signaling is unknown, but both MMP-8 and MMP-9 are markers of apical and periodontal disease [23].

An experimental study reported a significant elevation in the levels of MDA, which may be an indicator of oxidative stress [22]. The production of ROS after the activation of polymorphonuclear leukocytes may help in the formation of inflammatory lesions. The imbalance of oxidative species in the periapical part may also help the development of asymptomatic periapical lesions [24].

IL-6 and TNF-alpha mainly modulate the response of the cell during periodontal inflammation, persuade intracellular signaling, and alter the expression of the gene. Signaling molecules like chemokines, cytokines, and growth factors may be handled by the active form of MMPs, regulating their bioavailability and function [25-26]. The imbalance and abundance of cytokines and chemokines play an important role in the tissue changes during the development of periodontitis [27].

According to this study, the levels of SOD and GPx were significantly decreased in patients as compared to controls. The total antioxidant status indicates the ability of antioxidants to scavenge free radicals. The reduced activity of SOD and GPx may further the development of the lesion, resulting in oxidative stress [28]. Non-toxic levels of ROS increase proinflammatory mediators, and enzymes of the extracellular matrix take part in the destruction of apical tissue and the formation of apical lesions [26,29].

Our study was limited by the small sample size. Our findings should be validated in a study with a larger population.

## Conclusions

Increased levels of bone-resorbing mediators and proinflammatory cytokines (e.g., MMP-9, IL-6, C-reactive protein, TNF-alpha) have a well-established role in the course of progression of RC development. Moreover, an increase in the lipid peroxidation status (MDA), as well as a sharp rise in the reactive oxygen species (e.g., nitric oxide, advanced oxidation protein products, advanced glycation end-products), plays an important role in the pathogenesis of RC.
